# Viroid-like RNA-dependent RNA polymerase-encoding ambiviruses are abundant in complex fungi

**DOI:** 10.3389/fmicb.2023.1144003

**Published:** 2023-05-12

**Authors:** Li Chuin Chong, Chris Lauber

**Affiliations:** Institute for Experimental Virology, TWINCORE Centre for Experimental and Clinical Infection Research, a Joint Venture Between the Hannover Medical School (MHH) and the Helmholtz Centre for Infection Research (HZI), Hannover, Germany

**Keywords:** virus discovery, ambiviruses, human metatranscriptome, computational virology, viroid-like elements, fungal pathogen

## Abstract

Ambiviruses are hybrid infectious elements encoding the hallmark gene of RNA viruses, the RNA-dependent RNA polymerase, and self-cleaving RNA ribozymes found in many viroids. Ambiviruses are thought to be pathogens of fungi, although the majority of reported genomes have been identified in metatranscriptomes. Here, we present a comprehensive screen for ambiviruses in more than 46,500 fungal transcriptomes from the Sequence Read Archive (SRA). Our data-driven virus discovery approach identified more than 2,500 ambiviral sequences across the kingdom *Fungi* with a striking expansion in members of the phylum *Basidiomycota* representing the most complex fungal organisms. Our study unveils a large diversity of unknown ambiviruses with as little as 27% protein sequence identity to known members and sheds new light on the evolution of this distinct class of infectious agents with RNA genomes. No evidence for the presence of ambiviruses in human microbiomes was obtained from a comprehensive screen of respective metatranscriptomes available in the SRA.

## Introduction

Infectious genetic elements with RNA genomes (IGERs) encompass viroids and viroid-like RNAs as well as certain viruses. These agents have been demonstrated to cause a multitude of economically and medically important diseases despite major differences in genome size, genetic complexity, and life cycle between the various IGER classes and subclasses. The vast diversity of known and potentially unknown IGERs makes them ideal systems to study micro- and macro-evolutionary processes, the emergence of hybrid elements with features from difference IGER classes, and the origin(s) of life.

Viroids are the simplest form among known infectious pathogens, consisting of a single-stranded, covalently closed circular RNA genome of a few hundred nucleotides in length (Diener, 2001; Daròs et al., 2006). Although the known viroids do not encode proteins, they interact with the host via RNA structures to hijack the host transcription machinery for replication of their small RNA genomes. The members of different viroid families adopt distinct RNA structures, including branched as well as rod-shaped conformations (Giguère et al., 2014). Additional viroid-like IGERs include retroviroids, which integrate into the host genome with the help of a pararetrovirus (Daròs and Flores, 1995), circular satellite RNAs of plants, which require a helper virus for their replication and transmission (Bruening et al., 1991; Rao and Kalantidis, 2015), and animal-infecting ribozyviruses, including the important human pathogen hepatitis delta virus (HDV). Many viroids and viroid-like IGERs utilize IGER-encoded ribozymes, such as hammerhead ribozyme (HHR) or hairpin ribozyme (HPR), for cleavage of their multimeric replication products (Kos et al., 1986; Branch et al., 1988; Modahl et al., 2000; Ferre-D'Amare and Scott, 2010; Sureau and Negro, 2016; de la Peña et al., 2020; Wang, 2021). Known members of these classes have RNA genomes well below 2,000 nt.

Recently described ambiviruses employ considerably larger genomes in the range between 4,000 and 5,000 nt (Sutela et al., 2020; Forgia et al., 2021). Their circular RNA genomes exhibit unique features that make them hybrids between RNA viruses and viroids: they have two open reading frames (ORFs), one of which encodes an RNA-dependent RNA polymerase (RdRp) related to the RdRps of RNA viruses, while the function of the product encoded by the second ORF remains unknown. In addition, ambiviruses code for two HHR, HPR, or other types of ribozymes that are located close to each ORF’s C-terminal part in the non-protein-coding region of the genome. The two ORF-ribozyme pairs are encoded on opposite genome polarities. Ambiviruses are thought to infect fungi, although the large majority of ambivirus genomes have been discovered from metatranscriptomes (Sutela et al., 2020; Forgia et al., 2021, 2022). Due to the latter, a comprehensive and detailed ambivirus host distribution within and potentially also outside the kingdom *Fungi* is lacking. This paucity includes a description of the presence or absence of ambiviruses in the human mycobiome formed by fungal components of the microbiome which interact with both the bacterial microbiome and host immunity, and can influence pathophysiological processes in humans (Nguyen et al., 2015; Soret et al., 2020; Pérez, 2021; Zhang et al., 2022).

Here, we report the results of a screen for ambivirus genomes in almost 60,000 transcriptome projects of fungi and human microbiomes representing the full diversity available by the time of writing in the Sequence Read Archive (SRA). We discovered more than 2,500 viral sequences from 345 distinct ambiviruses and demonstrate an expansion of ambiviruses in the most complex fungal organisms from the phylum *Basidiomycota*. In general, our study offers new insights into the diversity and evolution of this distinct class of infectious agents.

## Results and discussion

**Figure 1:**
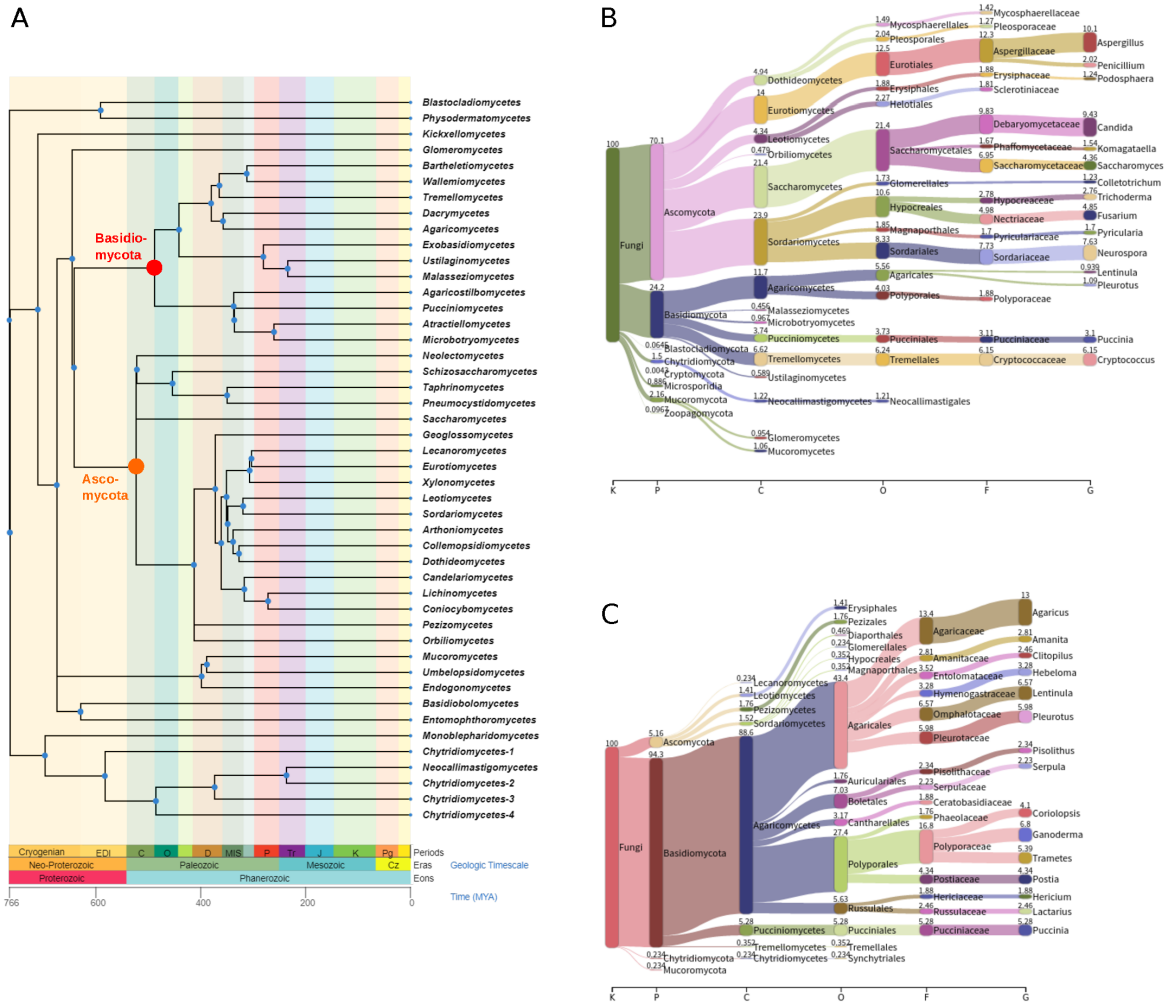
Phylogeny of fungi and taxonomic classification of fungal transcriptome experiments from the SRA. **(A)** The tree shows phylogenetic relationships of fungal classes and was obtained from Timetree 5 (http://timetree.org/). Branch lengths are in millions of years ago (MYA). The roots of the clades for *Ascomycota* and *Basidiomycota* are highlighted in orange and red, respectively. Sankey diagrams are shown for all 46,519 fungal SRA data sets that have been analyzed **(B)** and for those 853 of them in which ambivirus sequences have been discovered **(C)**. Numbers above colored bars show the percentage of SRA data sets at each taxonomic rank. Note the difference between relative frequencies of the phyla *Ascomycota* and *Basidiomycota* between the two diagrams. Taxonomic ranks shown are kingdom (K), phylum (P), class (C), order (O), family (F), and genus (G).

**Figure 2:**
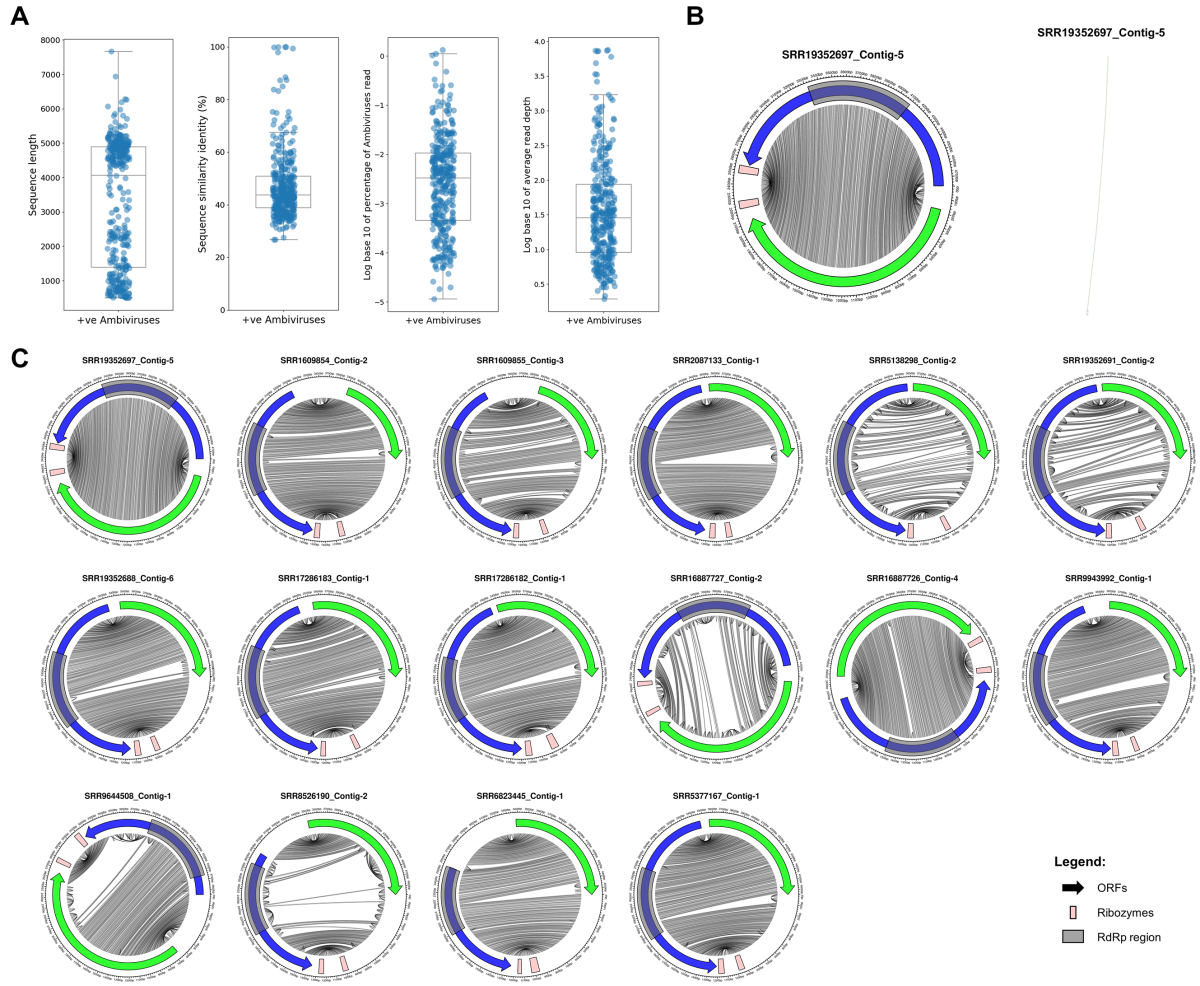
Assembled ambivirus genomes, their organization and RNA secondary structure of novel representatives. **(A)** Distributions of contig length, protein sequence similarity to the closest known ambivirus, percentage of viral reads and average read depth per viral genome position are shown for the 345 ambiviruses discovered in this study. **(B)** Left: The organization of a representative newly discovered ambivirus is shown. The accession number of the SRA experiment in which it has been identified is indicated. The ORF coding for the RdRp and the second largest ORF are shown as blue and green arrows, respectively. Light red rectangles indicate predicted ribozymes while gray rectangles indicate the RdRp region. Gray lines in the inner part of the circle connect bases in the genome that are predicted to pair in the RNA secondary structure. Right: RNA secondary structure conformation predicted by the RNAfold program from the ViennaRNA package for the ambivirus genome shown on the left. **(C)** Genomic organizations of 16 representatives with full-length circular RNA genomes; representation as in B. Genomes for which one half of the circle is fully connected to the other half correspond to rod-shaped secondary structure conformations while partially connected genomes indicate branched conformations.

We have applied a data-driven virus discovery (DDVD) approach (Lauber and Seitz, 2022) to screen a comprehensive set of 46,519 transcriptomes from the SRA representing the global diversity of fungi (Figure 1A) for the presence of ambivirus sequences. Our screening involved a sensitive sequence homology search in raw sequencing read data using a profile Hidden Markov Model (pHMM) of the ambivirus RdRp, which obtained hits against this ambivirus-specific pHMM in 853 SRA data sets. The subsequent seed-based genome assembly specifically targeted the sequences identified in the first stage and produced 2,588 contigs with significant sequence similarity to the ambiviral RdRp region including the well-conserved motifs A, B, and C (Gorbalenya et al., 2002; Bruenn, 2003). Removal of sequence redundancy by clustering the contigs at 90% nucleotide sequence identity and RdRp fragments shorter than 500 nt resulted in 345 unique ambivirus sequences of which 81 were full-length circular RNA genomes while the other assemblies represented incomplete genomes (Figure 2A; Supplementary Table S1). They have been retrieved from only 181 BioSamples in total (Supplementary Table S2), demonstrating the concurrent infection of individual fungi by several viruses. Although the viral reads typically constituted a minor fraction of the total amount of reads in a sequencing experiment (0.03% on average), the read depth per viral genome position was moderate to very high (273.7 on average; Figure 2A). The 345 discovered ambiviruses showed protein sequence identities to ambiviruses described in Forgia et al. (2022) and reference databases of 47.7% on average (range of 26.8–100%; Figure 2A), indicating that the majority of the ambiviruses discovered in this study are novel and that previous searches based on metatranscriptome analyses only revealed a fraction of the natural ambivirus diversity.

A strength of the SRA-based virus discovery approach is the availability of often detailed metadata, including host taxonomic information, for many of the underlying sequencing projects. We utilized this information (Supplementary Table S2) and mapped the fungal taxonomy of each sequencing project to the ambivirus sequences discovered from this project. Strikingly, although more than 70% of the analyzed SRA experiments studied fungi were from the phylum *Ascomycota* (Figure 1B), only very few ambiviruses (5.2%) were found in these fungi (Figure 1C). In sharp contrast, the large majority (94.3%) of the discovered ambiviruses were from the phylum *Basidiomycota*, which constituted only 24.2% of the analyzed SRA data sets. The difference between *Basidiomycota* and *Ascomycota* (Fisher’s exact test, *p* = 0) as well as *Mucoromycota* (*p* = 4.1*e*–28) and *Chytridiomycota* (*p* = 3.6*e*–19) was statistically highly significant, while no significant differences were observed between the other pairs of phyla (Supplementary Table S3).

*Ascomycota* species, including the model organism *Saccharomyces cerevisiae*, commonly (but not exclusively) reproduce asexually and are characterized by internal spore production in a sac-like structure called the ascus. Members from the *Basidiomycota* form spores externally by specialized cells called basidia, and sexual reproduction is considered to be more common among *Basidiomycota* species. It is tempting to speculate that the mode of reproduction may play a role in the spread of ambiviruses, a hypothesis that warrants further investigation, for instance via comparative infection experiments. Another factor of susceptibility to ambivirus infection might be linked to the higher complexity, in terms of cell cycle and multicellularity, of *Basidiomycota* species compared to those of other orders (Naranjo-Ortiz and Gabaldón, 2019). *Ascomycota* and *Basidiomycota* form two sister clades in the fungal tree of life (together building the most species-rich fungal subkingdom *Dikarya*) and constitute two relatively young lineages compared to the other fungal orders (Naranjo-Ortiz and Gabaldón, 2019), indicating that the observed expansion of ambiviruses in *Basidiomycota* was established after the split of the two orders.

The novel ambiviruses with full-length or near full-length genome sequences showed the expected genomic organization involving two open reading frames (ORFs) encoded in opposite reading directions (sense and antisense; Figures 2B,C). A self-cleaving hammerhead or hairpin ribozyme was found to be encoded near the C-terminus of each of the two ORFs (Figures 2B,C). We identified structural RNA motifs in 178 of the 345 ambivirus genomic sequences. When considering the top two hits per contig, the most frequent RNA structural motif was Hammerhead_3 ribozyme (*n* = 81, Rfam accession: RF00008), followed by Hairpin ribozyme (*n* = 77, RF00173) and Hairpin-meta1 ribozyme (*n* = 76, RF04190). Similar to other viroid-like elements, such as HDV, many of the ambivirus genomes are predicted to adopt a rod-shaped RNA secondary structure conformation (Figures 2B,C), while others show a branched conformation (Figure 2C).

**Figure 3:**
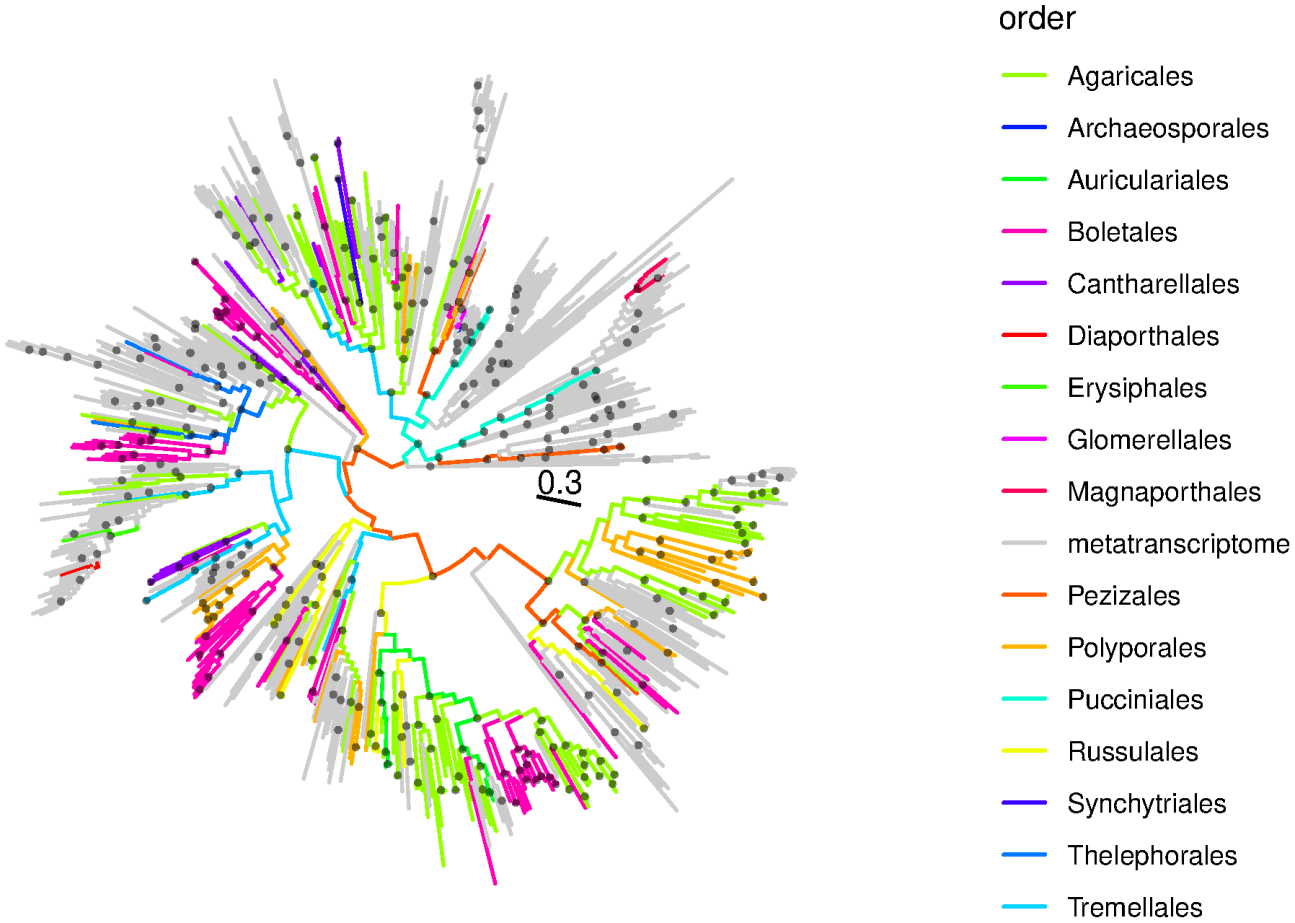
RdRp phylogeny of ambiviruses. Shown is a maximum likelihood RdRp phylogeny of ambiviruses in circular format. The tree has been mid-point pseudo-rooted. Branches are colored according to the fungal order of the SRA sequencing experiment from which the viral sequences were discovered; gray branches correspond to ambiviruses from metatranscriptome projects reported by Forgia et al. (2022) and used to create the HMM search profile used in this study. Black dots at internal nodes indicate branching events with SH-like support of 0.9 or better. The scale bar is in average amino acid substitutions per site.

We used RdRp protein sequences of previously described and newly discovered ambiviruses to reconstruct an ambivirus phylogeny (Figure 3). Viral groups of relatively low diversity were associated with specific fungal orders while the viral relationships predicted frequent cross-species transmissions at the macroevolutionary scale, as indicated by ambiviruses from a certain host order being scattered across the viral phylogeny (Figure 3). In addition, and in line with the viral sequence identity analysis presented above, the ambivirus phylogeny demonstrated that the majority of viruses discovered in this study constitute yet undescribed lineages. These undescribed lineages are distinct from known ambiviruses that are largely derived from metatranscriptomes (gray branches in Figure 3) and for which the host, therefore, remains unknown (Forgia et al., 2022; Lee et al., 2023). The discovery of 345 viral sequences from publicly available unprocessed sequencing archives reinforced the notion that data-driven virus discovery approaches (Lauber and Seitz, 2022) open new opportunities for studying the natural diversity and evolution of viruses, viroids, and other infectious agents that exist on our planet at unprecedented detail and depth. The DDVD approach is uncoupled from the collection, processing, and sequencing of biological samples and, thus, allows for projects of a scale that conventional virus discovery studies cannot compete with.

To investigate the potential relevance of ambiviruses to human health and disease, we performed a second screen of 12,694 human metatranscriptome projects from the SRA. This data set included samples from various human body sites. Out of the more than 12,000 experiments screened, there were only three data sets with ambivirus sequences fulfilling our hit criteria during the virus identification stage of our workflow (at least five read pairs identified with an *E*-value of 1*e*–5 or lower). Two of them were from the same study analyzing lung metatranscriptomes of patients with pneumonia and acute respiratory infections (SRA run accessions: SRR13677688 and SRR13677804). The third ambivirus-positive experiment (SRR5963935) was from a stool sample of a patient with Crohn’s disease. The very low number of identified ambivirus makes it very challenging to discriminate between origin of the ambiviruses sequences by infection of fungi from the human microbiome and origin by any source of contamination (Cobbin et al., 2021). In general, the virtual absence of ambiviruses from human metatranscriptomes suggests that these viroid-like elements do not present a major factor interacting with the fungal part of the human microbiome (Pérez, 2021). We note that we cannot fully exclude the possibility that the observed lack of ambiviral sequences in the data is caused by an absence or strong underrepresentation of fungal hosts in the human microbiome samples. Indeed, an inspection of a selected set of human metatranscriptomic data sets using the Taxonomy Analysis Tool at the NCBI/SRA website showed that no reads were classified as fungal for some of them. However, each of the SRA data sets inspected had a considerable fraction of “unidentified reads”, indicating that the reads cannot be assigned to any origin based on current reference sequences of known organisms. The proportion of unclassified reads varied greatly across data sets, and exceeded 98% in some cases (see for instance SRR935342). This observation reinforces the notion that our knowledge about the natural diversity of biological entities, both viral and cellular, remains incomplete.

## Conclusion

In summary, our study unveiled a large diversity of unknown ambivirus-like sequences in a large variety of fungi species. Future virus discovery efforts will show whether similar RdRp- and ribozyme-encoding hybrid elements exist in other hosts, including vertebrates and other animals. Studying the deep evolutionary relationships of this (and potentially other) distinct class(es) of sub-viral elements with ancient and extant RNA viruses may offer unprecedented insights into the emergence of RNA viruses and their hallmark RdRp protein.

## Methods

### Sequence Read Archive data and metadata

A list of all 46,519 publicly available transcriptome experiments representing the full diversity of the kingdom *Fungi* (except those of the model organisms *Saccharomyces cerevisiae* and *Schizosaccharomyces pombe*) in the NCBI SRA database was compiled as of October 2022. The following search query was performed to obtain the SRA run identifiers: ‘txid4751[Organism:exp] NOT txid4932[Organism:exp] NOT txid4896[Organism:exp] AND (cluster_public[prop] AND “biomol rna”[Properties])’. A list of all 12,694 human metatranscriptome experiments were compiled from the SRA database using the following search query: ‘txid1131769[Organism:exp] OR txid1504969[Organism:exp] OR txid1632839[Organism:exp] OR txid1633571[Organism:exp] OR txid1679718[Organism:exp] OR txid1712573[Organism:exp] OR txid1837932[Organism:exp] OR txid1842734[Organism:exp] OR txid2489051[Organism:exp] OR txid2705415[Organism:exp] OR txid408170[Organism:exp] OR txid433733[Organism:exp] OR txid447426[Organism:exp] OR txid539655[Organism:exp] OR txid646099[Organism:exp] AND (cluster_public[prop] AND “biomol rna”[Properties])’. SRA data were downloaded using the SRA Toolkit (Leinonen et al., 2011). The taxonomic identifier for each SRA data set was retrieved using the pysradb tool (Choudhary, 2019) while the taxonomic lineages information was fetched by the environment for tree exploration (ETE) toolkit (Huerta-Cepas et al., 2016). The host taxonomy information was reformatted into the Metaphlan2 format (Truong et al., 2015) using a customized script and the Pavian tool (Breitwieser and Salzberg, 2020) was used to produce Sankey diagrams.

### Sequence Read Archive-based virus discovery

The computational virus discovery workflow and its application to raw, unprocessed SRA data are described in previous studies (Lauber et al., 2017, 2021). The workflow is highly parallelized and was run on the high-performance computing cluster Taurus of the University of Technology (TU) Dresden. Here, we applied the Virushunter and Virusgatherer modules which screen a set of sequencing experiments from the SRA using one or several query pHMMs and perform a targeted, seed-based assembly of the identified sequencing experiments, respectively. The Virushunter performs a micro-assembly of sequencing read pairs identified in the pHMM search to create microcontigs that span the viral genome region covered by the query profile(s) (ambiviral RdRp in this study) or a part of that region. The Virusgatherer produces full-length or partial genome sequences depending on read coverage. Known ambiviruses previously discovered mostly in environmental metagenomes (Forgia et al., 2022) were used to construct a pHMM of ambivirus RdRp. We only considered hits for which at least five read pairs were identified with an E-value of 1e-5 or lower. Selected SRA data sets with high ambivirus read abundance were assembled *de novo* using SPAdes in RNA mode (Bankevich et al., 2012) to independently validate the results of the seed-based assembly. Full-length circular RNAs were identified using vdsearch (Lee et al., 2023).

The Virushunter and Virusgatherer tools as well as other code and further information are available on github: https://github.com/lauberlab/VirusHunterGatherer and https://github.com/lauberlab/ambivirus_discovery_paper.

### Open reading frame and RdRp identification

The presence of ORFs within the contig sequences was predicted using EMBOSS getorf (Rice et al., 2000). Only ORFs longer than 150 amino acids and inferred using the standard genetic code were considered for each circular RNA. Location of the RdRp was determined by comparing the *in silico* translated protein sequences encoded by the ORFs against the ambivirus RdRp profile with HMMER (Eddy, 2011).

### Ribozyme identification

The presence and genomic positions of ribozymes in ambivirus sequences were predicted using Infernal v1.1.4 (Nawrocki and Eddy, 2013) with the Rfam database (Kalvari et al., 2021). We considered hits with an E-value of 0.01 or lower.

### RNA secondary structure prediction

RNA secondary structure conformations of selected circular RNAs were predicted using RNAfold from the ViennaRNA package (Lorenz et al., 2011).

### Phylogenetic analysis

A multiple RdRp protein sequence alignment was computed using MAFFT v7.310 (Katoh and Standley, 2013) with options ‘--localpair --maxiterate 1000’, followed by manual curation. We only kept well-conserved RdRp alignment positions with less than 50% of gaps across all sequences, allowing the inclusion of RdRp fragments that contributed many of the gaps. We used ModelTest-NG v0.1.7 (Darriba et al., 2020) to determine the best-fitting amino acid substitution model, which was LG + G4 + I. Phylogenetic reconstruction was performed using PhyML v20120412 (Guindon et al., 2010). The tree was visualized using the ggtree R package (Yu et al., 2017).

A time-scaled phylogeny of fungal classes was obtained from TimeTree 5 (http://timetree.org/; Kumar et al., 2022).

### Statistical analysis

Contingency tables of the number of ambivirus-positive and -negative SRA experiments for a pair of host taxa to be compared and used Fisher’s exact test were compiled to assess the significance of differences. We used statistical functions (scipy.stats) in Python (Virtanen et al., 2020).

## Data availability statement

The original contributions presented in the study are included in the article/Supplementary material, further inquiries can be directed to the corresponding author.

## Author contributions

CL and LC performed experiments, analyzed the data and prepared figures. CL designed the study, supervised the project, and wrote the manuscript with contributions from LC. All authors contributed to the article and approved the submitted version.

## Funding

LC and CL are supported by the Project “Virological and immunological determinants of COVID-19 pathogenesis—lessons to get prepared for future pandemics (KA1-Co-02 ‘COVIPA’),” a grant from the Helmholtz Association‘s Initiative and Networking Fund. CL was supported by the Deutsche Forschungsgemeinschaft (DFG, German Research Foundation) under Germany’s Excellence Strategy—EXC 2155—project number 390874280. This publication is funded by the Deutsche Forschungsgemeinschaft (DFG) as part of the “Open Access Publikationskosten” program.

## Conflict of interest

The authors declare that the research was conducted in the absence of any commercial or financial relationships that could be construed as a potential conflict of interest.

## Publisher’s note

All claims expressed in this article are solely those of the authors and do not necessarily represent those of their affiliated organizations, or those of the publisher, the editors and the reviewers. Any product that may be evaluated in this article, or claim that may be made by its manufacturer, is not guaranteed or endorsed by the publisher.
